# A refined pH-dependent coarse-grained model for peptide structure prediction in aqueous solution

**DOI:** 10.3389/fbinf.2023.1113928

**Published:** 2023-01-16

**Authors:** Pierre Tufféry, Philippe Derreumaux

**Affiliations:** ^1^ Université Paris Cité, CNRS UMR 8251, INSERM U1133, Paris, France; ^2^ Université Paris Cité, CNRS UPR9080, Laboratoire de Biochimie Théorique, Institut de Biologie Physico-Chimique, Fondation Edmond de Rothschild, Paris, France; ^3^ Institut Universitaire de France (IUF), Paris, France

**Keywords:** peptide, structure, pH dependence, coarse grained models, prediction

## Abstract

**Introduction:** Peptides carry out diverse biological functions and the knowledge of the conformational ensemble of polypeptides in various experimental conditions is important for biological applications. All fast dedicated softwares perform well in aqueous solution at neutral pH.

**Methods:** In this study, we go one step beyond by combining the Debye-Hückel formalism for charged-charged amino acid interactions and a coarse-grained potential of the amino acids to treat pH and salt variations.

**Results:** Using the PEP-FOLD framework, we show that our approach performs as well as the machine-leaning AlphaFold2 and TrRosetta methods for 15 well-structured sequences, but shows significant improvement in structure prediction of six poly-charged amino acids and two sequences that have no homologous in the Protein Data Bank, expanding the range of possibilities for the understanding of peptide biological roles and the design of candidate therapeutic peptides.

## 1 Introduction

Peptides of less than 40 amino acids have diverse biological functions, acting as signaling entities in all domains of life, and targeting receptors or interfering with molecular interactions. Hormones and their bacterial mimetics (Fetissov et al., 2019), neuropeptides and their roles in neurodegenerative diseases (Ben-Shushan and Miller, 2021), antimicrobial peptides contribution to host defence (Mookherjee et al., 2020), and immunomodulatory peptides in the perspective of vaccine design (Pavlicevic et al., 2022) are some current directions motivating their study at a fundamental level. Due to their specific features, peptides have also gained interest as therapeutical agents (Muttenthaler et al., 2021), particularly to target protein-protein interactions (Cabri et al., 2021). They are also considered as having interest in the development of new functional biomimetic materials (Levin et al., 2020). Peptides have limitations though, as they can be highly flexible (Apostolopoulos et al., 2021), which motivate efforts to understand and predict their conformational energy landscapes.

Structure prediction of polypeptides with amino acid lengths up to 40 amino acids in aqueous solution can be performed by a series of methods including machine-learning approaches such as AlphaFold2 (Jumper et al., 2021), TrRosetta (Du et al., 2021), and APPTEST (Timmons and Hewage, 2021). Looking at AlphaFold2, which revolutionized structure prediction of single folded domain to a root-mean-square deviation (RMSD) accuracy of 0.2 nm, its capability lies on machine learning based on protein data bank (PDB) (Rose et al., 2012) templates, multiple sequence alignments, co-evolution rules and sophisticated algorithms to predict local backbone and side conformations, and side chain - side chain contact probability within distances bins. AlphaFold2 builds the protein by energy minimization using a protein-specific energy potential.

TrRosetta is basically similar to AlphaFold2. It builds the protein structure based on direct energy minimizations with a restrained Rosetta. The restraints include inter-residue distance and orientation distributions predicted by a deep neural network. Homologous templates are included in the network prediction to improve the accuracy further.

APPTEST also uses machine learning on the PDB structures with a chain length varying between 5 and 40 amino acids. APPTEST derives C*α*-C*α* and C*β*-C*β* distance restraints, and backbone dihedral restraints that are input of simulated annealing and energy minimization.

Other methods which are accessible by WEB-servers or can be downloaded include Rosetta (Bonneau et al., 2001), I-TASSER (Zhang, 2008), PepStrMod (Singh et al., 2015) and PEPFOLD (Shen et al., 2014; Lamiable et al., 2016). Rosetta is a fragment-assembly approach based on Monte Carlo simulation, a library of predicted nine and then three residues, and a coarse-grained model, followed by all-atom refinement. I-TASSER is a hierarchical approach that identifies structural templates from the PDB by multiple threading approaches, with full-length atomic models constructed by iterative template-based fragment assembly simulations.

The PEPstrMOD server predicts the tertiary structure of small peptides with sequence length varying between 7 and 25 residues. The prediction strategy is based on the realization that *β*-turn is an important feature of small peptides. Thus, the method uses both the regular secondary structure information predicted from PSIPRED and the *β*-turns information predicted from BetaTurns. The structure is further refined with energy minimization and molecular dynamic simulations.

PEP-FOLD2 is a fast accurate structure peptide approach based on the prediction of a profile of the structural alphabet of four amino acid lengths along the sequence, and a chain growth method based on the coarse-grained sOPEP2 model followed by Monte Carlo steps. It should be noted that PEP-FOLD2 is not free of learning as it uses an Support Vector Machine predictor relying on multiple sequence alignment. Of practical interest, during the time of this study, we could not access the APPTEST and PepStrMod servers. Also, TrRosetta cannot be applied to sequences with < 10 amino acids.

Overall, all these methods generate good models for well-structured peptides at pH 7 in aqueous solution because most structures deposited in the PDB from nuclear magnetic resonance (NMR) and X-ray diffraction experiments were determined at neutral pH, and the PDB contains close to 200,000 structures as of 30 October 2022.

These methods face, however, two current limitations: correct conformational ensemble sampling of intrinsically disordered peptides or proteins (IDPs) which lack stable secondary and tertiary structures, and accurate conformational ensemble prediction of peptides as a function of pH and salt conditions. The first issue has motivated the development of new force fields, such as CHARMM36m-TIP3P modified (Huang et al., 2017), AMBER99SB-DISP (Robustelli et al., 2018) and many others (Nguyen and Derreumaux, 2020). The current approach to address the impact of pH variations is to perform your own extensive molecular dynamics and replica exchange molecular dynamics simulations at your desired pH. Alternatively one can use pH-replica exchange molecular dynamics using a discrete protonation method (Sabri Dashti et al., 2012) or all-atom and coarse-grained continuous constant pH molecular dynamics (CpHMD) methods (Barroso da Silva et al., 2016; Huang et al., 2016; Aho et al., 2022). Accurate and fast peptide structure predictions at different pH and salt conditions are the objectives of the present study.

The organization of this paper is as follows. In section 2, we present an extension of the coarse-grained, sOPEP2, force field to integrate Debye-Hückel charge interactions as a function of pH and salt concentrations. Next, we present the TrRosetta, AlphaFold2 and PEP-FOLD with and without Debye-Hückel protocols and the analysis methods. In section 3, we present the results of structure predictions of six poly-charged peptides as a function of pH and compare them to experimental circular dichroism (CD) data, and the predicted models obtained by TrRosetta and AlphaFold2. The charged polypeptides are particularly interesting to assemble the sOPEP2 interactions and the Debye-Hückel charge interactions. This is followed by the prediction of 15 ordered peptides, which have NMR structures resolved at a pH varying from 2 to 8. We finish this section on the prediction of four peptides for which low-resolution experimental data and topological descriptions are available. Finally section 4 summarizes our findings.

## 2 Methods

### 2.1 The sOPEP2 force field

The sOPEP2 potential, to be used in a discrete space, originates from the OPEP potential which uses an explicit representation of the backbone (N, H, C*α*, N and H atoms) and one bead for each side chain, whose position to C*α* and Van der Walls radius depend on the amino acid type (Maupetit et al., 2007; Sterpone et al., 2014). The sOPEP2 is expressed as a sum of local, non-bonded and hydrogen-bond (H-bond) terms, with all parameters described in (Binette et al., 2022).

$$E=E_{\mathrm{local}}+E_{\mathrm{nonbonded}}+E_{H-bond}$$ (1)

Since the geometry in PEP-FOLD is mainly imposed by the superimposition of the discrete structural alphabet (SA) letters, the local contributions are restricted to a simple flat-bottomed quadratic potential to described the energy associated with dihedral angles *ϕ* and *ψ*, described by:

$$E\left(\phi_{i}\right)=\epsilon_{\phi }{\left(\phi_{i}-\phi_{0\text{\_}sc\text{\_}i}\right)}^{2}$$ (2)

where *ϕ*_0_*sc*_*i*_ = *ϕ* within the interval $\left[\phi_{low\text{\_}sc\text{\_}i},\phi_{high\text{\_}sc\text{\_}i}\right]$ and *ϕ*_0_*sc*_*i*_ = min(*ϕ* − *ϕ*_*low*_*sc*_*i*_, *ϕ* − *ϕ*_*high*_*sc*_*i*_) outside of the interval *ϕ*_*low*_*sc*_*i*_ and *ϕ*_*high*_*sc*_*i*_ are specific to each amino acid type (Binette et al., 2022).

Non-bonded interactions corresponding to repulsion/attraction effects are described using the Mie potential (Mie, 1903) given by:

$$E_{vdw\text{\_}ij}=\epsilon_{ij}\times \left[\frac{m}{n-m}{\left(\frac{r_{ij}^{0}}{r_{ij}}\right)}^{n}-\frac{n}{n-m}{\left(\frac{r_{ij}^{0}}{r_{ij}}\right)}^{m}\right]$$ (3)

where *ϵ*_*ij*_ is the potential depth and $r_{ij}^{0}$ is the position of the potential minimum function of atomic types for *i* and *j*. The combination of exponents, *n* and *m*, gives the relationship between the position of the potential minimum (*r* ^0^) and the position where it is zero (*gR*0):

$$gR0={\left(\frac{m}{n}\right)}^{\frac{1}{n-m}}r_{0}$$ (4)

Hydrogen bonds are considered explicitly, using a combination of two types of contributions:

$$E_{H-bond}=E_{\mathrm{HBpairwise}}+E_{HBcoop}$$ (5)

where $E_{HB_{\mathrm{pairwise}}}$ corresponds to the two-body contributions of all hydrogen bonds between residue (i) and residue (j), characterized by the hydrogen/acceptor distance *r*_*ij*_ and the donor/hydrogen/acceptor angle *α*_*ij*_:

$$E_{\mathrm{HBpairwise}}\left(r_{ij},\alpha_{ij}\right)=\epsilon_{\alpha }^{HB}\underset{ij,j=i+4}{\sum }\mu \left(r_{ij}\right)\cdot \nu \left(\alpha_{ij}\right)+\epsilon_{\beta }^{HB}\underset{ij,j>4}{\sum }\mu \left(r_{ij}\right)\cdot \nu \left(\alpha_{ij}\right)$$ (6)

$$\mu \left(r_{ij}\right)=\epsilon_{ij}\cdot \left[5{\left(\frac{\sigma }{r_{ij}}\right)}^{12}-6{\left(\frac{\sigma }{r_{ij}}\right)}^{10}\right]$$ (7)

$$\nu \left(\alpha_{ij}\right)=\left\{\begin{array}{cc} \cos\left(\alpha_{ij}\right)\; & \text{if }\alpha_{ij}>90{}^{\circ}\\ 0\; & \text{otherwise} \end{array}\right.$$ (8)

where *σ* = 0.18 nm is the position of the potential minimum and *ϵ* is the potential depth. We distinguish between *α*-helix-like hydrogen bonds defined by O(i)-H(i+4) and other hydrogen bonds. Hydrogen bonds between a pair of residues separated by less than four amino acids are not considered.

$E_{HB_{\mathrm{coop}}}$ involves four-body interactions involving pairs of hydrogen bonds (between residues (i) and (j) and residues (k) and (l)), so as to stabilize secondary structure motifs. The cooperation energy is given by the following equations:

$$E_{\mathrm{HBcoop}}\left(r_{ij},r_{kl}\right)=\epsilon_{\alpha }^{\mathrm{coop}}\sum C\left(r_{ij},r_{kl}\right)\times \Delta \left(ijkl\right)+\overset{\mathrm{coop}}{\underset{\beta }{\epsilon }}\sum C\left(r_{ij},r_{kl}\right)\times \Delta^{\prime }\left(ijkl\right)$$ (9)

$$C\left(r_{ij},r_{kl}\right)=\exp\left(-0.5{\left(r_{ij}-\sigma \right)}^{2}\right)\cdot \exp\left(-0.5{\left(r_{kl}-\sigma \right)}^{2}\right)$$ (10)

$$\Delta \left(ijkl\right)=\left\{\begin{array}{cc} 1\; & \text{if }\left(k,l\right)=\left(i+1,j+1\right)\\ 0\; & \text{otherwise} \end{array}\right.$$ (11)

$$\Delta^{\prime }\left(ijkl\right)=\left\{\begin{array}{cc} 1\; & \text{if }\left(k,l\right)=\left(i+2,j-2\right)\\ \; & \text{or }\left(k,l\right)=\left(i+2,j+2\right)\\ 0\; & \text{otherwise} \end{array}\right.$$ (12)

### 2.2 Debye-Hückel charge interactions

The new sOPEP version introduces the possibility to consider pH-dependent charge interactions, using the Debye-Hückel formulation (Debye and Hückel, 1923).

$$E_{DH_{ij}}=\left(q_{i}*q_{j}*e^{-r_{ij}/l_{DH}}\right)/\left(\epsilon \left(r_{ij}\right)*r_{ij}\right)$$ (13)

where *q*_*i*_ and *q*_*j*_ correspond to the charge of particles *i* and *j*, *j* > *i* + 1, respectively, *r*_*ij*_ is the distance between the particles, *l*_*DH*_ is the Debye length that depends of the ionic strength of the solvent, and *ϵ*(*r*_*ij*_) is the dielectric constant that depends on the distance between the charges:

$$\epsilon \left(r\right)=D_{w}-\left(D_{w}-D_{p}\right)\left(s^{2}r^{2}+D_{p}sr+D_{p}\right)e^{-sr}/D_{p}$$ (14)

where *D*_*w*_ is the dielectric constant of water, *D*_*p*_ is the dielectric constant inside a protein, and *s* is the slope of the sigmoidal function. In practice, we used values of 78, 2 and 0.6 for *D*_*w*_, *D*_*p*_ and *s*, respectively, as stated in (Iwaoka et al., 2020).

Since the sOPEP representation does not include all-atom side chains, but charges associated with particles of heterogeneous sizes, it is necessary to shift the energy curve to have energy values compatible with those of the Mie formulation. For each pair of particle, we shifted the distance using:

$r_{ij}^{SH}=r_{ij}+shift_{ij}$ and we evaluated $E_{DH_{ij}}$ using $r_{ij}^{SH}$ except for *ϵ*(*r*), where the unshifted distance is used.

**FIGURE 1:**
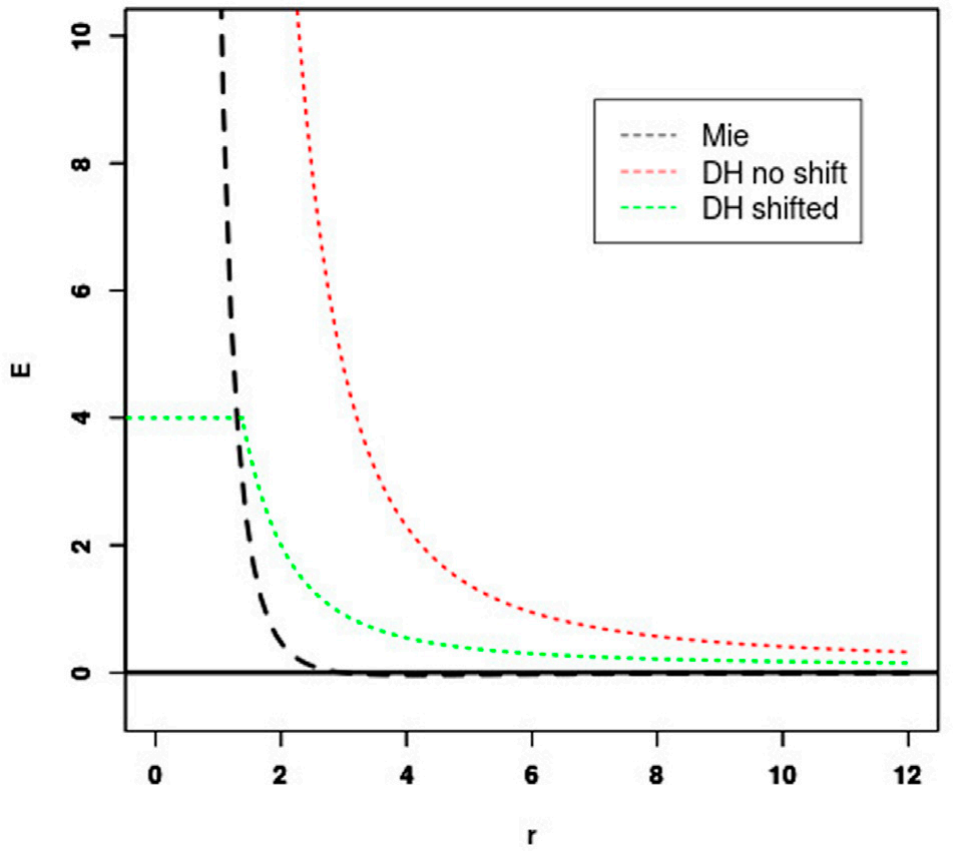
Fitting Debye-Hückel (DH) energy to Mie potential. The shift of the unshifted DH potential (red) is set so that the Mie (black) and shifted DH potential (green) cross for some energy threshold (4 kcal/mol in this case).

Shift values were adjusted for *r* such as *E*_*vdw*_*r*_ = *k*, $E_{DH_{r}}=E_{vdw\text{\_}r}$ , as illustrated in Figure 1. In practice, we found that values of *k* on the order of 4 kcal/mol are convenient, and the Debye-Hückel energy was truncated to $E_{DH_{r}}$ to avoid redundancy with the Mie potential. Also, as sOPEP2 side-chain side-chain potential already includes some of the interactions between the charged residues, the Mie potential is restricted to only the repulsive part for charged side chains.

Charges were assigned to particles depending on the pH using pKa values of 3.9, 4.2, 6.0, 10.5 and 12.5 for ASP, GLU, HIS, LYS and ARG side chains, respectively, and 9.0 and 2.0 for the N-terminal *α*-ammonium and C-terminal *α*-carboxyl groups, respectively. Note that it is possible to consider blocking the extremities using acetyl and N-methyl on the N-terminus and C-terminus groups, respectively, in which case no charge is assigned to the extremity.

Finally, we have considered weighting differently the electrostatic contributions depending on the separation of the amino acids in the sequence. In our experience, best results were obtained using a weight of 10 for residue separation of less than 7 amino acids and a weight of 2, otherwise.

### 2.3 PEP-FOLD, TrRosetta and AlphaFold2 protocols and analysis

Our validation test set includes a total of 25 peptides as described in Section 3. For each peptide, we performed two PEP-FOLD simulations, one TrRosetta simulation which uses PDB templates and homologous sequences, and one AlphaFold2 simulation in its standard version using three recycles, template information, and AMBER refinement. Both TrRosetta and AlphaFold2 simulations return five models that we considered equiprobable. The PEP-FOLD simulations without Debye-Hückel (referred to as PF-noDH), and with Debye-Hückel (PF-DH) generated 200 models each. We selected the representative models of the five best clusters identified among the 200 generated models based on their rankings using sOPEP2 energies - i.e. the standard PEP-FOLD model selection - for PF-noDH and the sum of sOPEP2 and Debye-Hückel energies for PF-DH.

We have considered 15 peptides for which a PDB structure is available. These correspond to peptides previously studied during PEP-FOLD development and new peptides with their structures released after 1 September 2019, and determined in pure aqueous environment. The predicted models of the 15 peptides were evaluated by computing the CAD-score (Olechnovič et al., 2013). The reported CAD-score corresponds to the largest value of the cross CAD-scores between the five predicted models and all NMR structures. Following our previous work, if the CAD-score calculated on the backbone atoms is > 0.60, the model is associated with largely correct secondary structure prediction, otherwise if it is > 0.65 the model is correct in terms of secondary and tertiary structures. For the poly-charged peptides, we also computed their secondary structure contents using STRIDE program (Frishman and Argos, 1995). For the four sequences free of any NMR structure, we compared their predicted and experimental topologies.

## 3 Results and discussion

### 3.1 Predicted models of poly-charged peptides

For the simulations of the six poly-charged peptides, namely (EK)15, (EK)5, (H)30, (E)15, (K)15 and (R)25, we calculated the alpha-helix, coil and turn contents averaged over the five models of each method and compared with circular dichroism (CD) experiments. It is to be noted that by default TrRosetta, AlphaFold2 and PF-noDH only perform simulations at neutral pH. CD values are not available at all pH varying from 3 to 13. We report, however, on the pH-dependent conformations using PF-DH. Results are summarized in Table 1.

**TABLE 1:** Structural impact of pH variation on poly-charged peptides.

	Experiment			PF-noDH			PF-DH			AlphaFold2			TrRosetta		
	Exp. Blck	pH	CD	*α*	Coil	*β*-turn	*α*	Coil	*β*-turn	*α*	Coil	*β*-turn	*α*	Coil	*β*-turn
		3					28	69	3						
(EK)15	-	7.4	*β*-turn	94	6	0	0	85	15	97	3	0	97	3	0
		11					24	68	8						
		3					0	100	0						
(EK)5	-	7.4	coil	90	10	0	0	68	32	54	10	36	68	26	6
		11					0	78	22						
(H)30	-	5	coil				0	100	0						
		7.4	aggregation	75	5	20		95	5	0	81	19	97	3	0
(K)15	Ace, Nmt.	3.6	0% *α*				0	100	0						
		7.4	0% *α*	93	7	0	0	100	0	73	8	19	93	7	0
		11.2-13	83.7% *α*				93	7	0						
		3–3.6	ND-42% *α*				93	7	0						
(E)15	Ace, Nmt.	4.2	75% *α*				93	7	0						
		7.4	0% *α*	93	7	0	0	100	0	93	7	0	93	7	0
(R)25	-	5.7	50% coil				0	100	0						
		7.4	ND	96	4	0	0	100	0	77	4	19	96	4	0
		11.3	50% coil 15%*α*				0	100	0						
		13					96	0	0						

Results are presented for PEP-FOLD, without and with Debye-Hueckel (PF-noDH, PF-DH), AlphaFold2 and TrRosetta. Experimental information about the blocking of extremities (Exp. Blck.) using acetyl (Ace) or N-methyl (Nmt), pH and Circular Dichroism (CD) data, is reported. ND, stands for not determined.

TrRosetta, AlphaFold2 and PF-noDH have a very high propensity to report alpha-helical conformations for the six polypeptides at pH 7.4, the exception being (H)30, with alpha-content varying from 54% to 97%, while CD displays only coil or beta-turn signals. For instance, for (EK)5, TrRosetta reports 68% helix and 26% coil, AlphaFold2 reports 54% helix and 10% coil and PF-noDH reports 90% helix and 10% coil. Only PF-DH is able to predict the CD coil character of (EK)5 with 68% coil and 32% turn.

PF-DH is the single method to predict 85% coil and 15% turn consistent at pH 7.4 with the beta-turn CD signal of (EK)15 (Smith et al., 2020), and PF-DH predicts 100% coil at pH 5 consistent with the coil CD signal of (H)30 (Jesus, 2020). There is strong experimental evidence that (H)30 polymerizes at pH 7.4 forming beta-sheets. At this pH, PF-noDH and TrRosetta predict strong helical conformations, while PF-DH and AlphaFold2 predict a random coil, with contents of 95% and 81%, respectively.

**FIGURE 2:**
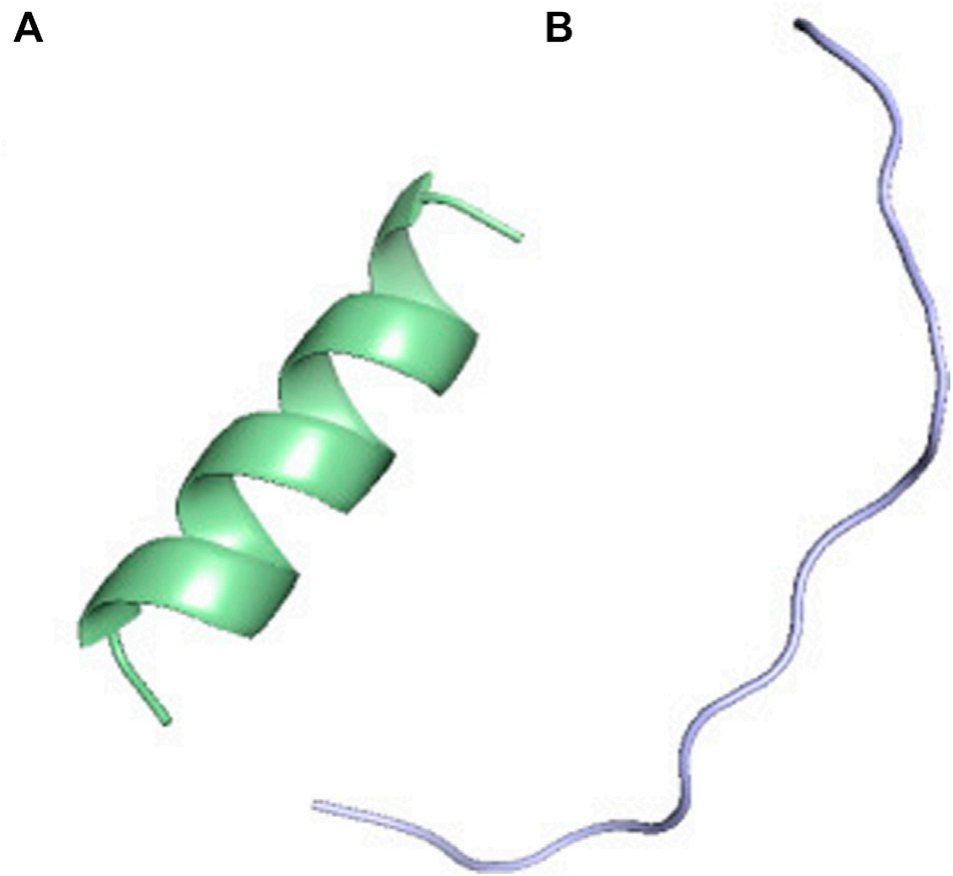
PF-DH Conformational ensemble of (K)15 as a function of pH. **(A)** pH 7.4 **(B)** pH 13. Only the lowest energy model (rank 1) is depicted.

**FIGURE 3:**
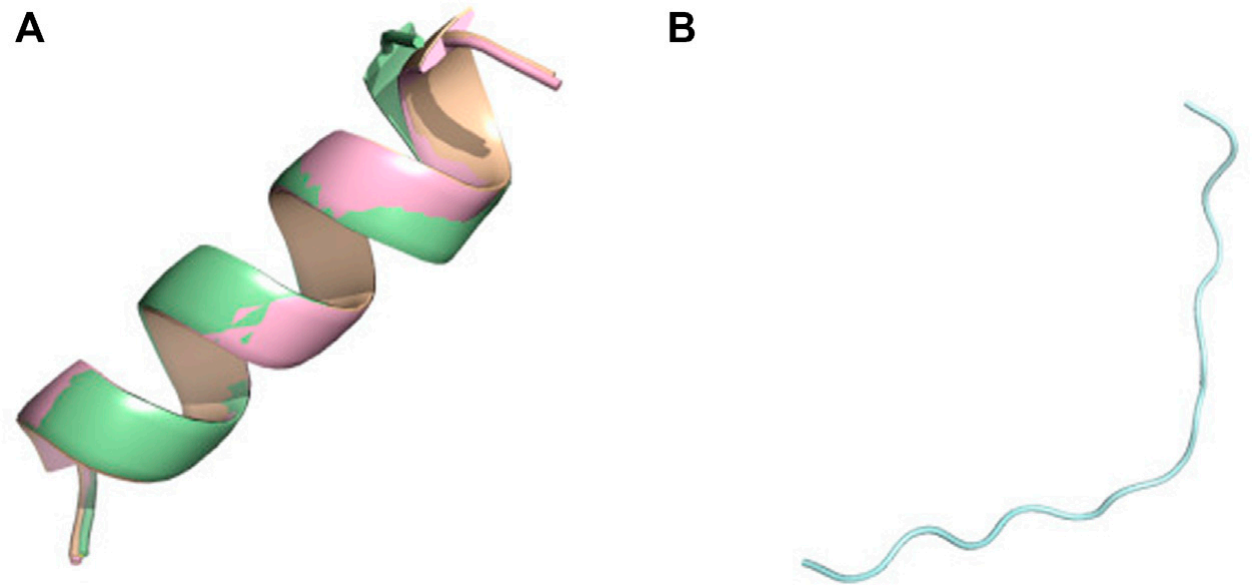
Conformational ensemble of (E)15 at pH7.4. **(A)** PF-noDH, AlphaFold2 and TrRosetta **(B)** PF-DH. Only the lowest energy (rank 1) model is depicted.

The polypeptides (K)15 and (E)15 are particularly interesting because the alpha-helix content changes inversely with the pH. As observed by CD, the helical content of (K)15 increases with pH, while the helical content of (E)15 decreases with pH (Batys et al., 2020). (K)15 have 0% helix at pH 3.6 and 83.7% helix at pH 11–13 by CD. PF-DH finds 0% helix at pH 3.6 and 93% helix at pH 11–13 (Figure 2). In contrast to (K)15, (E)15 (Figure 3) have 42% helix at pH 3.6 and 0% helix at pH 11–13 by CD. PF-DH finds 93% helix at pH 3.6 and 100% coil at pH 11–13.

The conformation ensemble of (R)25 is predicted to have 50% coil and 31% beta-sheet at pH 5.7 and have 51% coil and 21% beta-sheet at pH 11.3 by CD (Morga et al., 2022). PF-DH predicts 100% coil, independently of the pH values. Its performance is however much better than those of PF-noDH, AlphaFold2 and TrRosetta which predict a high helical signal varying from 77% to 96%.

Overall, the structure predictions of the six polypeptides at neutral pH (7.4) give quite different contents of the secondary structure using PF-noDH and PF-DH, with PF-noDH behaving and failing like AlphaFold2 and TrRosetta predictions. This result emphasizes the role of the Debye-Hückel charged-charged interactions when treating poly-charged peptides. The results also demonstrate that the learning stage of the local conformations in PEP-FOLD performed from structures at neutral pH can be counterbalanced by the force field, making possible to explore new conformations depending on the pH. In contrast, AlphaFold2 and TrRosetta rely on homologous structures and multiple sequence alignements. Since neither is available for poly-charged peptides, it is normal for both predictors to fail. But surprisingly, the LDDT (local distance difference test) metric predicted by both methods is, on average, very high ( > 80%) for all amino acids of the six poly-charged peptides.

It is important to emphasize that in this study, we assume the standard pka values of charged amino acids irrespective of the amino acid composition of the peptides and the conformations of the peptides. This is a strong limitation of our current approach. Determining the pka values of charged amino acids in protein structures has motivated the development of many theoretical methods (Sabri Dashti et al., 2012; Barroso da Silva et al., 2016; Huang et al., 2016; Aho et al., 2022). To illustrate the variation of the pka values, we used the H++ server which is based on classical continuum electrostatics and basic statistical mechanisms (Anandakrishnan et al., 2012). Using (K)15, we found pka values ranging from 10.1 to 9.4 (versus 10.5 in our model); using (R)25, we found pka ranging from 9.6 to 11.6 in one conformation, and from 10.9 to 11.7 in another conformation (versus 12.5 in our model), and using (H)30, we found pka variations from 4.7 to 6.3 (versus 6.0 in our model). Clearly this change of pka of the amino acids will impact the equilibrium conformations of PF-DH.

### 3.2 Predicted models of polypeptides with NMR structures

The experimental information of each well-ordered peptide, given in Table 2, includes the amino acid length varying from 8 to 35 amino acids, the number of NMR models, the WDC (well-defined rigid core) according to the PDB, the topology, the pH varying from 4.3 to 7, the ionic strength varying from 0 to 150 mM NaCl, the blocking of the extremities and the amino acid sequence.

**TABLE 2:** Peptide set.

Target	L	# mod.	WDC	Topo.	pH	ion.Str	Exp. Blck.	Sequence
6mi9	19	10	3–19	*α* distort.	4.3	0	Nmet.	PMARNKILGKILRKIAAFK
6j9p	12	10	2–12	*α*	5	0	-	RRLIRLILRLLR
1fsd	28	41	3–25	*β*_2_ *α*	5	0	-	QQYTAKIKGRTFRNEKELRDFIEKFKGR
1j4m	14	1	ND	*β*_2_	5	0	-	RGKWTYNGITYEGR
1le1	12	20	2–11	*β*_2_	5.5	0	Nmet.	SWTWENGKWTWK
6nm3	8	5	ND	*α*-like	5.8	0	Nmet.	RKIWWWWL
6svc	35	20	2–35	*β*_3_	6	150	-	SKLPPGWEKRMSRNSGRVYYFNHITNASQFERPSG
2evq	12	43	2–10	*β*_2_	6	20	-	KTWNPATGKWTE
1egs	9	20	1–9	*β*_2_-like	6.5	0	Ace, Nmet.	TKSAGGIVL
6r2x	25	15	9–20	C*α*	6.5	148	-	FETLRGDERILSILRHQNLLKELQD
7b2f	31	20	6–21	*αCα*	6.5	100	-	MNNNELTSLPLAERKRLLELAKAAKLSRQHY
6s0n	9	20	ND	*α*-like	6.8	1	-	QDVNTAVAW
7li2	22	20	1–22	*β*_2_-like	7	0	-	AGTMRVTYPDGQKPGQSDVEKD
1wbr	17	32	1–16	*αC*	7	0	Ace, Nmet.	QAERMSQIKRLLSEKKT
1pgbF	16	1	ND	*β*_2_	7	0	-	GEWTYDDATKTFTVTE
pep17	17	-		*α*	2	0	-	ETGTKAELLAKYEATHK
pep38	38	-		*αTα*	3.6	20	-	DWLKARVEQELQALEARGTDSNAELRAMEAKLKAEIQK
pep10	11	-		*β*_2_	4.3	0	-	IYSNSDGWTWT
tau fragment	17	-		*β*_2_	7	0	-	DNIKHVPGGGSVQIVYK

For each peptide with an experimental structure available we specify its PDB, identifier (PDB), size (L), the number of NMR, models available (# mod.), its well defined core according to the PDB (WDC), its topology (topo.); and the experimental conditions including the pH, the ionic strenght (ion. Str.) and the presence of extra groups to block the N terminus (acetyl) and C terminus (N methyl) (Exp. Blck.), and the amino acid sequence. Four additional peptides without deposited structures but for which information exists in the literature are reported at the bottom of the table.

Table 3 reports on the CAD-scores using the full sequences and the rigid cores of each of the 15 peptides using the four methods. Note we give the results of PF-noDH, AlphaFold2 and TrRosetta, because these methods which are pH independent are used irrespective of the experimental pH conditions.

**TABLE 3:** Performance prediction for structured peptides.

	Exp.		PF-noDH			PF-DH			AlphaFold2			TrRosetta		
Target	Topo	pH	Topo	Full	WDC	Topo	Full	WDC	Topo	Full	WDC	Topo	Full	WDC
6mi9	*α*	4.3	*α*	0.85	0.86	*α*, *α*-like	0.84	0.86	*α*	0.83	0.86	*α*	0.84	0.84
6j9p	*α*	5	*α*	0.75	0.77	C*α*	0.70	0.71	*α*	0.74	0.76	*α*	0.74	0.75
1fsd	*β*_2_ *α*	5	*α*C*α*	0.62	0.63	*β*2*α*	0.66	0.69	*β*_2_ *α*	0.73	0.76	*β*_2_ *α*	0.71	0.73
1j4m	*β*_2_	5	*β*_2_	0.67	0.67	*β*_2_	0.76	0.76	*β*_2_	0.74	0.74	*β*_2_	0.73	0.73
1le1	*β*_2_	5.5	*β*_2_	0.76	0.8	*β*_2_	0.75	0.75	*β*_2_	0.83	0.87	*β*_2_	0.8	0.79
6nm3	*α*-like	5.8	*α*	0.72	0.72	*α*,unstr	0.73	0.73	coil	0.59	0.59	-	-	-
6svc	*β*_3_	6	*β*_3_	0.66	0.66	*β*_3_	0.70	0.70	*β*_3_	0.79	0.8	*β*_3_	0.78	0.79
2evq	*β*_2_	6	*β*_2_	0.84	0.86	*β*_2_	0.82	0.86	*β*_2_	0.89	0.93	*β*_2_	0.8	0.79
1egs	*β*_2_-like	6.5	*β*_2_, *β*_2_-like	0.71	0.71	*β*_2_-like	0.70	0.70	coil	0.55	0.55	-	-	-
6r2x	C*α*	6.5	C*α*	0.75	0.9	C*α*	0.74	0.91	*α*	0.8	0.92	C*α*	0.8	0.9
7b2f	*α*C*α*	6.5	*αC*, *α*	0.77	0.82	*αCα*	0.79	0.84	*αCα*	0.79	0.84	*αCα*	0.77	0.83
6s0n	*α*-like	6.8	*α*	0.76	0.76	*α*	0.79	0.78	coil	0.67	0.67	-	-	-
7li2	*β*_2_-like	7	*β*_2_, *β*_2_-like	0.62	0.62	*β*_2_, *β*_2_-like	0.64	0.64	coil	0.6	0.6	coil	0.58	0.58
1wbr	*α*C	7	*α*	0.69	0.7	*α*	0.70	0.70	*α*	0.69	0.7	*α*	0.68	0.69
1pgbF	*β*_2_	7	*β*_2_	0.77	0.77	*β*_2_	0.79	0.78	*β*_2_, *ext* − *untr*	0.83	0.83	*β*_2_	0.91	0.91
MEAN				0.73	0.75		0.74	0.76		0.74	0.76		0.76	0.78
STDEV				0.07	0.09		0.06	0.08		0.10	0.12		0.08	0.09
MEDIAN				0.75	0.76		0.74	0.75		0.74	0.76		0.77	0.78

For each structure, we report a short description of the topology of the 5 best models, and the CAD-score values (see methods) obtained for PEP-FOLD without and with Debye-Huckel (PF-noDH, PF-DH), AlphaFold2 and TrRosetta. Note that TrRosetta is not functional for amino acid lengths < 10.

The first striking result is that (the mean, standard deviation and median) values of the CAD-scores averaged over the 15 peptides are nearly identical for the four methods using both the full sequences or the rigid cores. They reach (0.73, 0.07, 0.75) for PF-noDH, (0.74, 0.10, 0.74) for AlphaFold2, (0.74, 0.06, 0.74) for PF-DH and (0.76, 0.08, 0.77) for TrRosetta using the full sequences. Similar trends are observed considering the well-defined rigid cores, the average CAD-scores being of 0.75, 0.76, 0.76 and 0.78 for PF-noDH, PF-DH, AlphaFold2 and TrRosetta, respectively.

**FIGURE 4:**
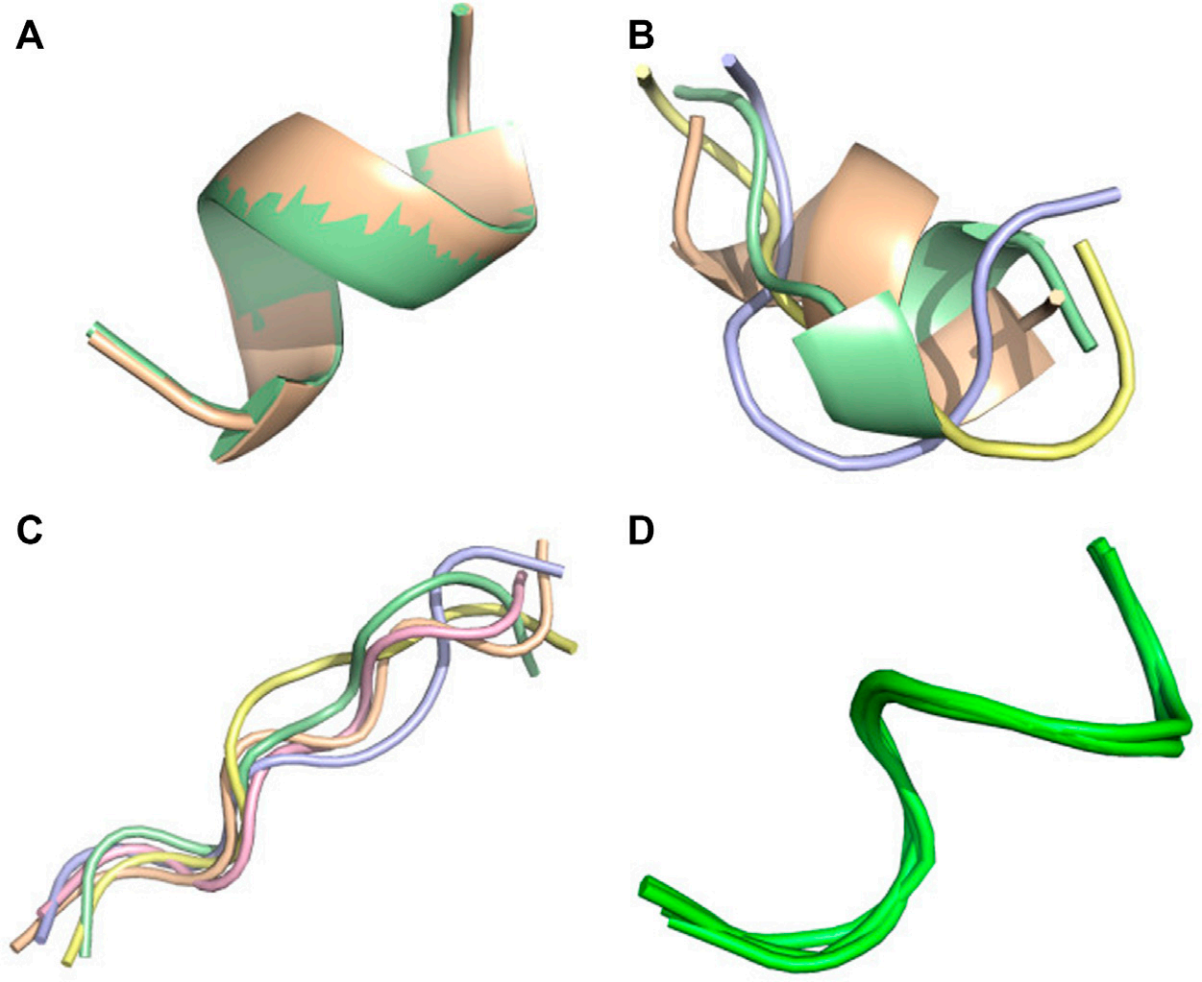
Conformational ensemble of 6 nm3. **(A)** PF-noDH, **(B)** PF-DH at pH 4.3, **(C)** AlphaFold2, **(D)** NMR structure. For A, B and C, the 5 predicted models are depicted. For D, all models provided in the PDB are depicted.

**FIGURE 5:**
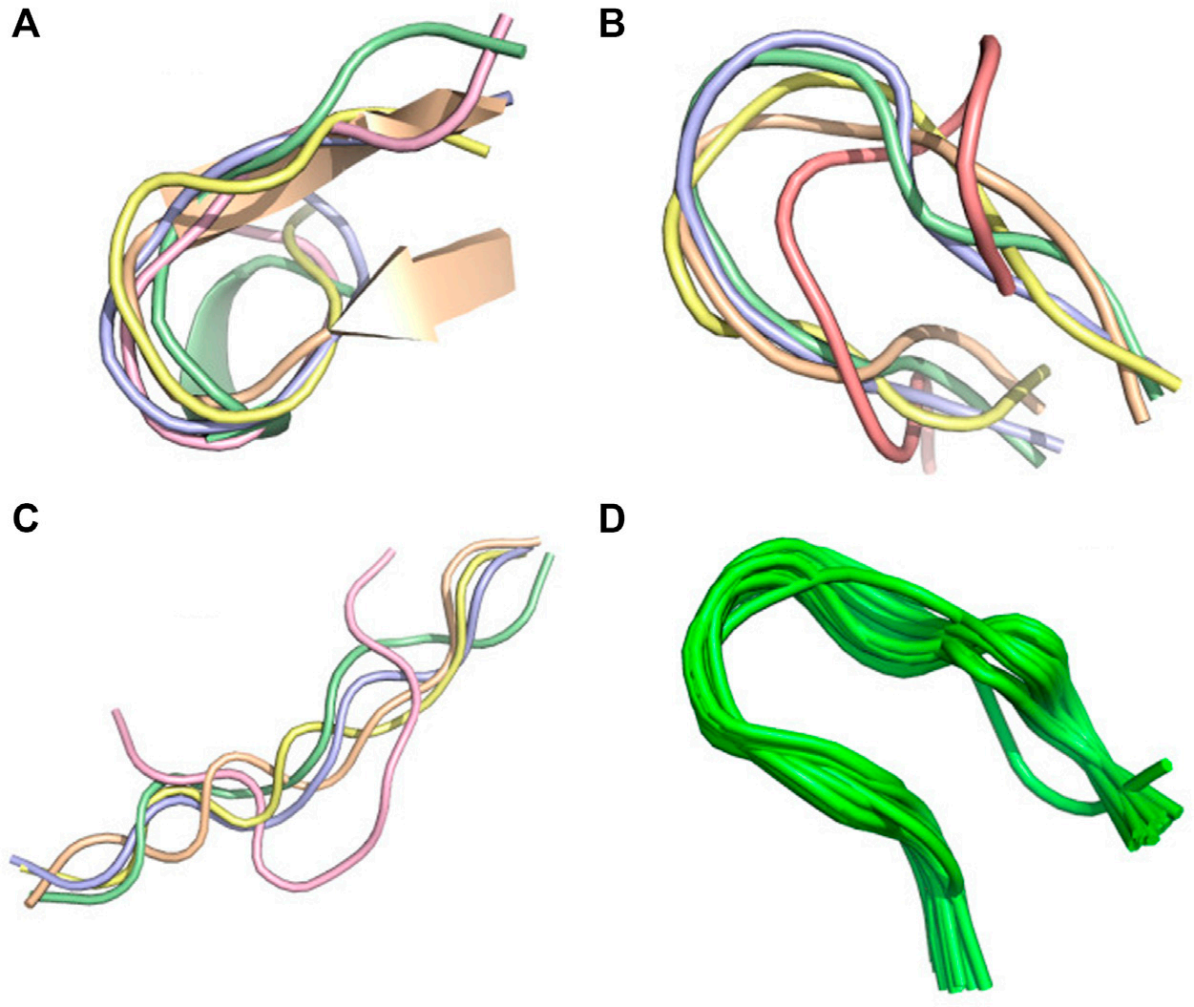
Conformational ensemble of 1egs. **(A)** PF-noDH, **(B)** PF-DH at pH 4.3, **(C)** AlphaFold2, **(D)** NMR structure. For A, B and C, the 5 predicted models are depicted. For D, all models provided in the PDB are depicted.

**FIGURE 6:**
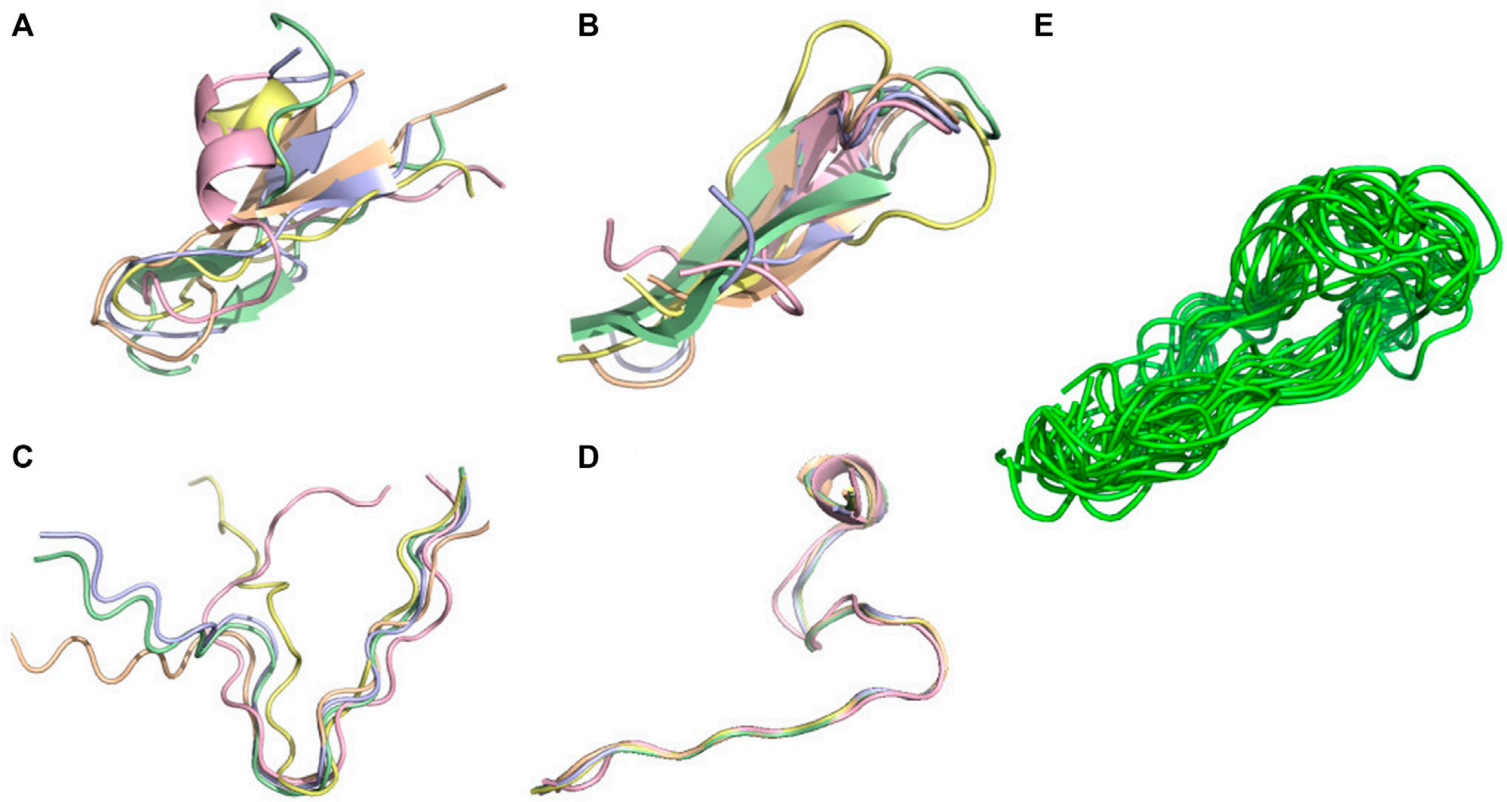
Conformational ensemble of 7li2. **(A)** PF-noDH, **(B)** PF-DH at pH 4.3, **(C)** AlphaFold2, **(D)** TrRosetta, **(E)** NMR structure. For A, B, C and D the 5 predicted models are depicted. For E, all models provided in the PDB are depicted.

The second result is that PF-noDH and PF-DH do not predict any low quality models (CAD-score < 0.6), while AlphaFold2 produces CAD-scores of 0.59, 0.55 and 0.6 for the three targets 6nm3,1egs and 7li2 (Figures 4–6, respectively). It has to be noted that the structures of these three peptides were solved at pH 5.8, 6.5 and 7. This low score results in differences between the experimental and predicted topologies. Experimentally, 6nm3 adopts a helical-like conformation, 1egs adopts a beta2-like conformation and 7li2 adopts a beta-2 like conformation. Of note, a beta-2 like conformation has the topology of a beta-hairpin but lacks the H-bond network.

For these three systems, AlphaFold2 predicts an extended-unstructured conformation. The 7li2 target is also problematic for TrRosetta, as it is the single system with a CAD-score < 0.6, namely 0.58 leading to an extended-unstructured conformation. Inversely, TrRosetta is the best method to predict the beta-hairpin of 1pgbF (Barducci et al., 2011) with a CAD-score of 0.91 versus 0.83 with AlphaFold2 and 0.79 with PF-DH.

The third result is related to the performance of PF-DH with respect to PF-noDH, which provides evidence that the weights of the Debye-Huckel salt bridge interactions are consistent with the weights of sOPEP2 interactions. It was far from being evident that the addition of charges at extremities and charged amino acids in the core of the sequences would not change the quality of the models for pH varying between 2.9 and 7. The number of titratable amino acids varies from 1–2 (1le1, 1egs - 6nm3, 6evq), 4 for 1j4m, 5 for 6j9p and 1pgbF, 6 for 6mi9, 7 for 1wbr and 7li2, 9 for 6r2x, 10 for 6svc and 7b2f, to 12 for 1fsd. The results also show that the pH-independent PEP-FOLD version and the pH-dependent PEP-FOLD version perform similarly for peptides containing charged, hydrophilic and hydrophobic amino acids.

Finally, using NMR structures as a reference, a very recent study benchmarked the accuracy of AlphaFold2 in predicting 588 peptide structures between 10 and 40 amino acids, including soluble peptides, membrane-associated peptides, and disulfide-rich peptides (McDonald et al., 2023). Although the study ignores pH conditions and the presence of the membrane, AlphaFold2 can be used for the modeling of peptide structures anticipated to have a well-defined secondary structure. AlphaFold2 is particularly successful in the prediction of alpha-helical membrane-associated peptides and disulfide-rich peptides, but also shows some shortcomings in predicting phi and psi angles. It was found that AlphaFold2 performs at least as well if not better than TrRosetta and PEP-FOLD using our 2016 set of parameters.

### 3.3 Predicted models of polypeptides without any NMR structures

The last four peptides have been discussed in literature in terms of topological features without delivering any NMR structure. Their sequences are given at the bottom of Table 2.

**FIGURE 7:**
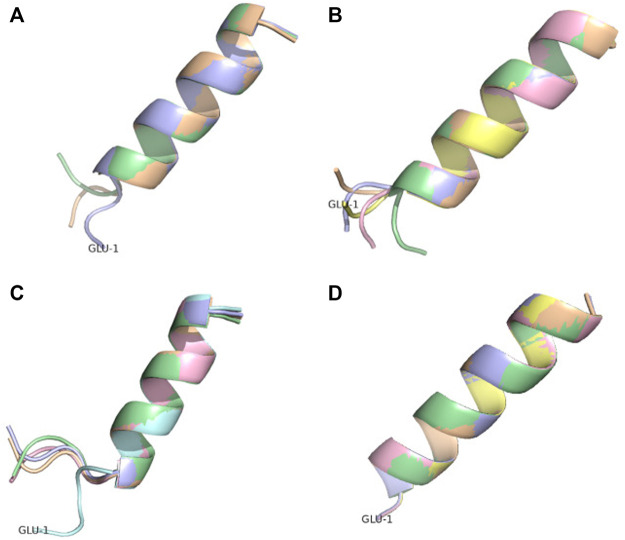
Conformational ensemble of pep17. **(A)** PF-noDH, **(B)** PF-DH—pH 2, **(C)** AlphaFold2 and **(D)** TrRosetta. For each method, the 5 predicted models are depicted.

Two peptides are rather well described by all four methods. Pep17 has been shown as a stable monomeric helix at pH two using CD and NMR experiments (Bradley et al., 1990). PF-noDH, PH-DH at pH 2 and AlphaFold2 predict a helical conformation with a frayed N-terminus, while TrRosetta predicts a full helical conformation (Figure 7). Pep38 determined experimentally as a helix-turn-helix at pH 3.6 (Fezoui et al., 1997) is also well reproduced by the four methods.

**FIGURE 8:**
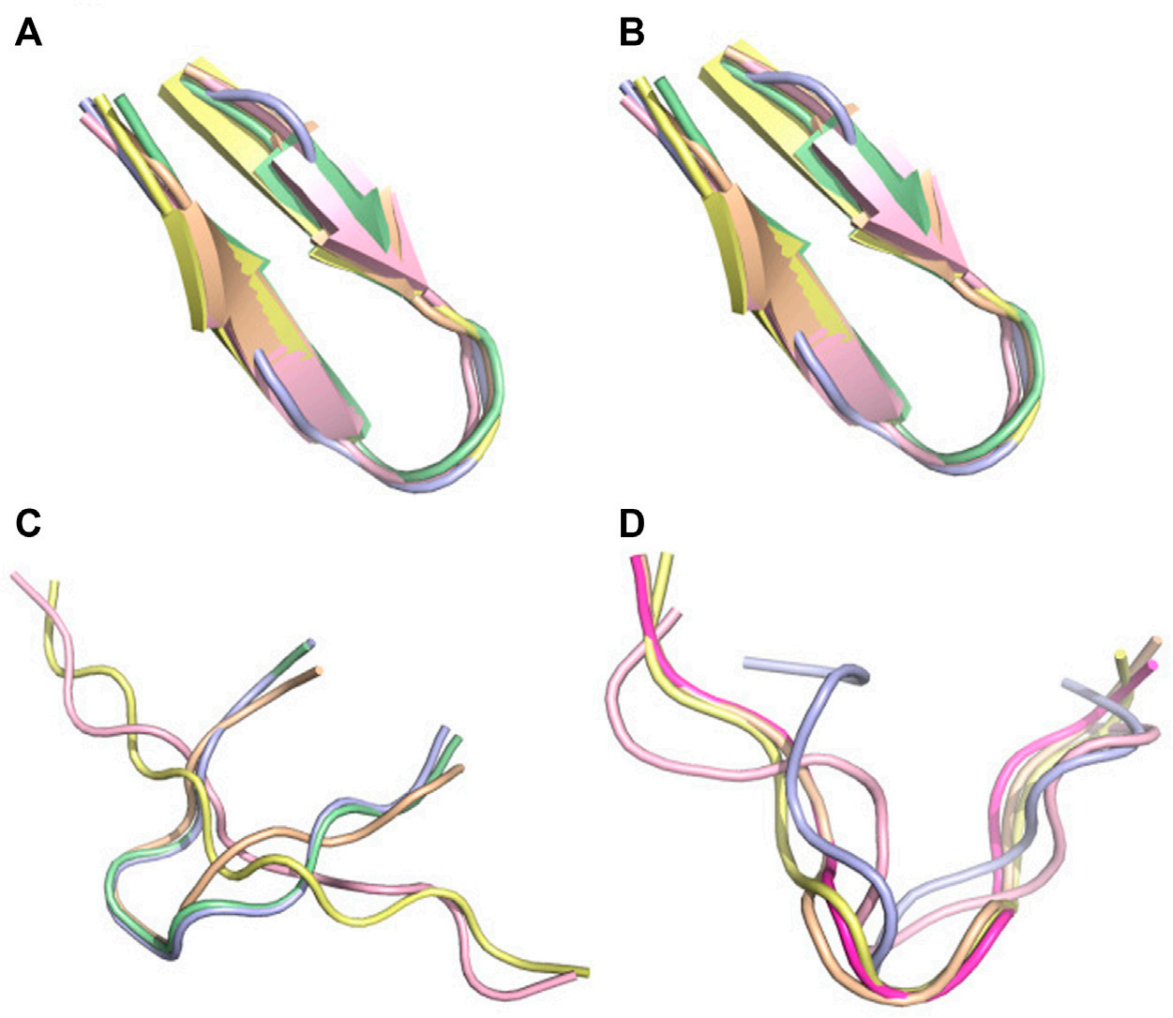
Conformational ensemble of pep10. **(A)** PF-noDH, **(B)** PF-DH at pH 4.3, **(C)** AlphaFold2, **(D)** TrRosetta. For each method, the 5 predicted models are depicted.

There are two cases, where AlphaFold2 and TrRosetta fail to produce the experimental data. The first peptide is pep10 which is described experimentally by an ensemble of distinct transient beta-hairpins at pH 4.3(Alba et al., 1997). It is described as an unstructured turn-like conformation by TrRosetta (Figure 8D), and an ensemble of extended and beta2-like conformations by AlphaFold2 (Figure 8C). In contrast, PF-noDH and PF-DH predict well a beta-hairpin conformation (Figures 8A,B).

**FIGURE 9:**
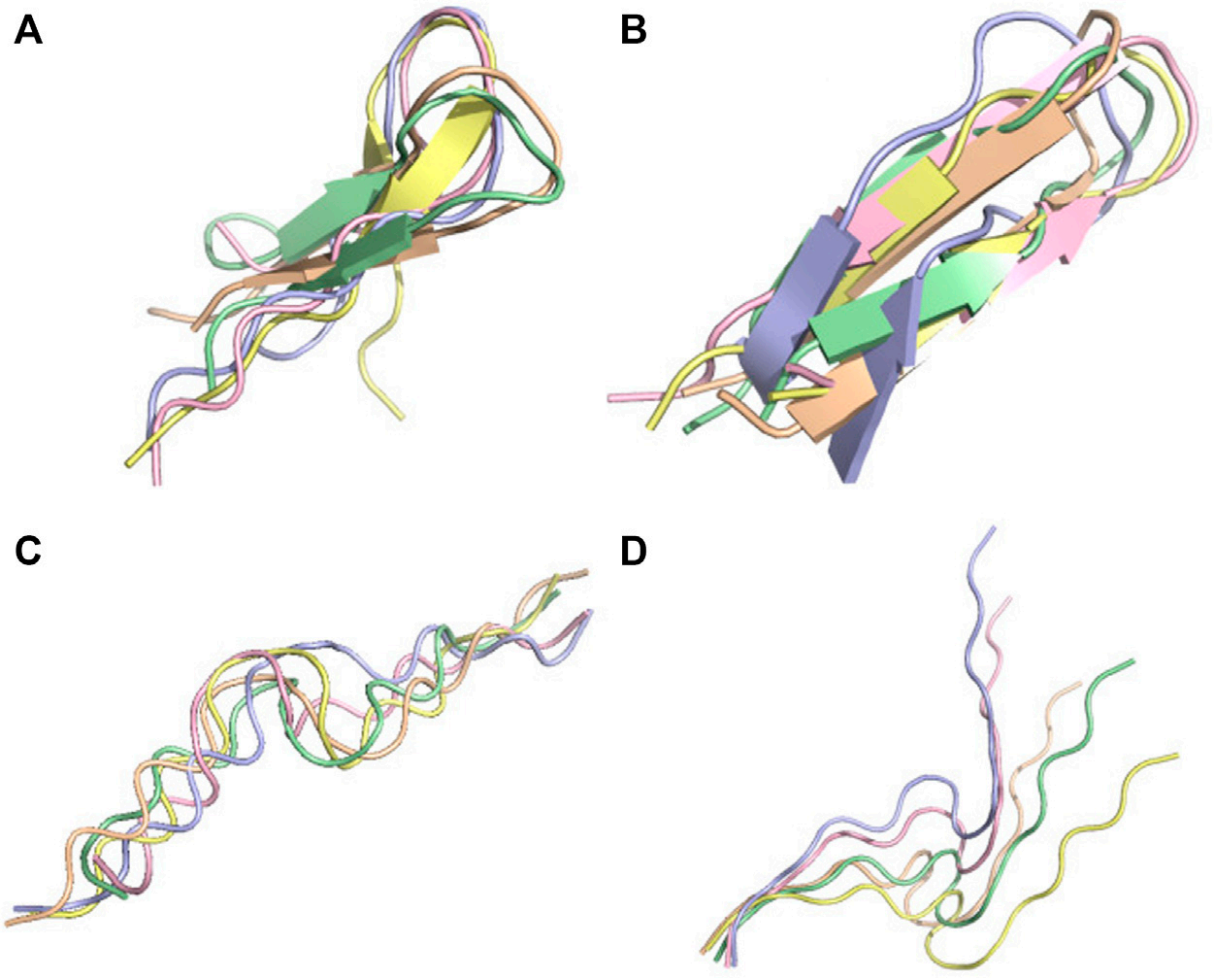
Conformation ensemble of tau-fragment at pH 7. **(A)** PF-noDH, **(B)** PF-DH, **(C)** AlphaFold2 and **(D)** TrRosetta. For each method, the 5 predicted models are depicted.

The second peptide is the tau fragment encompassing residues 295–306 containing the aggregation-prone PHF6 motif (306–311). Using cross-linking mass-spectrometry, *ab initio* Rosetta (Ovchinnikov et al., 2018), and CS-Rosetta which leveraged available chemical shifts (Lange et al., 2012) for the tau repeat spanning residues 243–365, the tau fragment 295–306 was predicted as a beta-hairpin at pH 7 (Chen et al., 2019). PF-noDH and PF-DH predict the same conformation (Figures 9A, B). In contrast, AlphaFold2 predicts extended conformations (Figure 9C), and surprisingly TrRosetta finds a random coil conformation (Figure 9D).

Overall, this small set of peptides provides evidence of some limitations of AlphaFold2 and TrRosetta when the target does not have an homologous sequence in the PDB.

## 4 Conclusion

Integrating pH variation effects to a coarse-grained model, where the side chains are represented by one single bead, is an important step toward accurate polypeptide structure prediction in aqueous solution, as coarse-graining with various granularities (de Vries and Zacharias, 2013; Sieradzan et al., 2022), enhance sampling. This task has been performed by combining a Debye-Hückel formalism for charged - charged side chain interactions and the sOPEP2 potential. By using a total of 25 peptides of amino acid lengths varying between 7 and 38 amino acids, this study provides evidence that PF-noDH, PF-DH, AlphaFold2 and TrRosetta perform similarly on peptides deposited in the Protein data Bank, but PF-DH outperforms the two recent machine-learning methods for poly-charged peptides, and peptides for which homologous sequences are not deposited in the PDB. Of note, our new formulation takes into account the impact of salt concentration variations, but we could not identify from the literature any case reporting a conformation change upon ionic strength variation.

Overall this work is one step towards peptide structure prediction in mimicking *in vivo* conditions. We are currently working on IDP’s in aqueous solution and *de novo* structure prediction of peptides at the surface of two-dimensional cell membranes.

## Data Availability

The original contributions presented in the study are included in the article/Supplementary Material, further inquiries can be directed to the corresponding author.
